# Total Flavone of Rhododendron Improves Cerebral Ischemia Injury by Activating Vascular TRPV4 to Induce Endothelium-Derived Hyperpolarizing Factor-Mediated Responses

**DOI:** 10.1155/2018/8919867

**Published:** 2018-10-11

**Authors:** Jun Han, Hang-Hang Xu, Xiao-Long Chen, Hao-Ran Hu, Kun-Mei Hu, Zhi-Wu Chen, Guo-Wei He

**Affiliations:** ^1^Drug Research and Development Center, School of Pharmacy, Third-Grade Pharmacology Laboratory of State, Administration of Traditional Chinese Medicine, Anhui Provincial Engineering Research Center for Polysaccharide Drugs, Wannan Medical College, Wuhu, Anhui, China; ^2^Department of Pharmacology, School of Basic Medicine, Anhui Medical University, Hefei, Anhui 230031, China; ^3^Department of Cardiovascular Surgery and Center for Basic Medical Research, TEDA International Cardiovascular Hospital, Chinese Academy of Medical Sciences and Peking Union Medical College, Tianjin, China; ^4^Department of Surgery, Oregon Health and Science University, Portland, OR, USA

## Abstract

**Background:**

Total flavonoids of Rhododendron (TFR) is extracted from Rhododendron, a herbal medicine widely used in China. The main components are flavone compounds such as warfarin, rutin, quercetin, and hyperoside. We investigated the role of TRPV4 channel in the TFR induced endothelium-dependent hyperpolarizing factor- (EDHF-) mediated responses against ischemia/reperfusion injury (IR) in cerebral IR (CIR) rats.

**Methods:**

The morphological changes of cerebral cortex, the relaxation of cerebral basal artery (CBA), and cell membrane potential recording were studied in CIR rats. The outward potassium current in smooth muscle cell was recorded by whole-cell patch clamp recording. The protein expression of TRPV4, SKca, and IKca was determined. Confocal laser was used to measure the Ca^2+^ fluorescence intensity.

**Results:**

After treatment with TFR, the number of pyramidal cells in brain tissue increased and the number of empty or lightly stained cells decreased and these effects were eliminated by using HC-067047, Apamin, or TRAM-34. TFR induced and EDHF-mediated dilatation and hyperpolarization in CBA were also attenuated by using these inhibitors. The increased outward current density elicited by TFR in acutely isolated CBA smooth muscle cells was abolished by using TRAM-34 and Apamin. TFR upregulated the protein expression of TRPV4, SKca, and IKca that was also eliminated by these inhibitors. Laser scanning showed that the increased mean fluorescence intensity of Ca^2+^ by CIR was decreased by using TFR and that this effect was again eliminated by the above inhibitors.

**Conclusions:**

We conclude that in the CBA of the CIR rats the protective effect of TFR on ischemic cerebrovascular injury may be related to the activation of the TRPV4 in both endothelium and smooth muscle by increasing its expression and activity. The activation of TRPV4 channel in the endothelium may be linked to the opening of endothelial IKca/SKca channels that induces EDHF-mediated relaxation and hyperpolarization in the smooth muscle cell. In addition, the activation of TRPV4 in the smooth muscle cell in CBA may be linked with the activation of BK_Ca_ channel through a TRPV4-dependent pathway, reduce Ca^2+^ concentration in the cell, and relaxes the vessel. These findings may form a new therapeutic target for protection of ischemic brain injury and facilitate the use of Chinese medicine in brain protection.

## 1. Background

Ischemic cerebral vascular disease, such as ischemic stroke, has high incidence, causing high disability and mortality rate. It is often caused by cerebral arterial embolism or thrombosis, leading to transient or persistent decrease in the blood flow of the cerebral artery and resulting in irreversible changes in the structure and function of the brain. Clinically, ischemic cerebrovascular disease usually occurs at the basilar artery (CBA) and other cerebral arteries. In addition, spasm of the artery may also result in a sharp decrease of the cerebral blood flow, causing ischemia. Vascular tension changes caused by cerebrovascular contracting and relaxing factors play a pivotal role in ischemic cerebrovascular disease [1], including endothelium-derived relaxing factors such as prostacyclin (PGI_2_) [2], nitric oxide (NO) [3], and endothelium-derived hyperpolarizing factor (EDHF) [4–7]. EDHF plays an important role in physiological and pathological processes. Particularly, in traumatic brain injury and other pathological conditions, EDHF plays a key role in regulation of cerebral blood flow [8, 9] and is considered to be a promising new target for treatment of cardiovascular and cerebrovascular diseases [10, 11].

Mammalian transient receptor potential (TRP) channels are grouped into six members. TRP vanilloid channel (TRPV) is a subfamily of the TRP family. TRPV4 is distributed in vascular endothelial cells, smooth muscle cells, neurons, and glial cells. The opening of TRPV4 leads to Ca^2 +^ influx and triggers a series of Ca^2 +^ dependent physiological reactions, such as releasing of acetylcholine (ACh) and other media [12] and opening of intermediate conductance Kca (IKca or K_Ca_3.1) and small conductance Kca (SKca or K_Ca_2.3) channels [13]. Further, TRPV4 may be involved in the Ca^2+^ entering into the cells, triggering endothelial activation, and promoting EDHF-induced vascular relaxation response [14].

Total flavones of Rhododendron (TFR) is the effective flavonoid component extracted from Rhododendron flowers and its main ingredients are matteucinol, quercetin, rutin, hyperoside, and flavonoids. TFR has a positive effect on anticerebral ischemic injury by reducing the area of cerebral infarction, alleviating cerebral edema and cerebral cell apoptosis [15, 16]. Our previous studies have demonstrated that TFR induces EDHF-mediated vasodilatation and smooth muscle cell membrane hyperpolarization in the cerebral basilar artery of rats with cerebral ischemia-reperfusion (CIR) injury and that the effect of TFR on brain blood vessels in rats was inhibited by the nonspecific TRPV4 blocker ruthenium red (RR) [17]. Similar to above-mentioned, studies have shown that activation of TRPV4 may promote the opening of SKca and IKca channels [18, 19] that are widely distributed in the cardiovascular and cerebrovascular system and related to diseases.

The present study was aimed at exploring the relationship between the protective effect of TFR on ischemic brain injury and the function of TRPV4, SKca, and IKca channels with exclusion of the role of NO and PGI_2_ under both* in vivo* and* in vitro* situations in rat models of global cerebral ischemia and reperfusion in order to further explore the new mechanism and strategies for prevention of cerebral ischemia injury.

## 2. Materials and Methods

### 2.1. Animals

Male Sprague-Dawley rats weighing 230~270g, 8 weeks old, were procured from Nanjing Qinglongshan Experimental Animal Company (Certificate No. Scxk 2013-0006, Nanjing, China). The rats were adaptive feeding for one week. The indoor temperature was (23±2)°C and the relative humidity was 55%~60% with natural light. The animals were free to drink and eat. All animal studies and surgical procedures were conformed to the regulations defined by the Ethical Committee of Wannan Medical College, which were strictly in line with the Guide for the Care and Use of Laboratory Animals (US National Research Council, 2011).

### 2.2. Drugs and Reagents

Total flavones of Rhododendron simsii Planch (TFR) with content of flavones greater than 85% were supplied by Hefei Heyuan Medicine Technology Limited Company (Hefei, China). Nissl staining solution, N-nitro-L-arginine-methyl-ester, Dithiothreitol, BCA protein assay kit, GAPDH antibody, Rabbit IgG, and Mouse IgG were purchased from Beyotime Institute of Biotechnology (Haimen, China). The KCNN4 antibody was purchased from Thermo Fisher Scientific (Waltham, USA). The KCNN3 antibody was purchased from Abcam (Cambridge, UK). HC-067047, TRAM-34, Apamin, indomethacin, TRPV4 antibody, and papain were purchased from Sigma (St. Louis, MO, USA). Calcium fluorescence probe Fluo-3/AM was purchased from Dojindo (Shanghai, China).

### 2.3. Primary Instrument

Model 550 microplate reader, miniprotein electrophoresis system, and miniprotein transfer membrane system were purchased from BIO-RAD (California, USA). KD paraffin microtome was purchased from Shanghai fourth medical instrument factory (Shanghai, China). OLYMPUS bx-41 microscope was purchased from OLYMPUS (Tokyo, Japan). AlC-CWB numerical control constant temperature circulating water tank was purchased from Shanghai Alcott Biotech Co., Ltd. (Shanghai, China). Multichannel microsampling system was purchased from Inbio Life Science Instrument Co., Ltd. (Wuhan, China). Glass electrode drawing instrument was purchased from MDI (USA). Leica TCS Sp8 confocal laser scanning microscope was purchased from Leica (Germany).

### 2.4. Establishment of CIR Rat Model

The rats were initially anesthetized with 4 % isoflurane during induction and then maintained with 2 % isoflurane in a mixture of 30 % O_2_ and 70 % N_2_O. The rats were fixed in prone position, and then cut in the center of the posterior neck for a ~2cm incision. The bilateral pterygoid foramen of the first cervical vertebra was exposed. The electrocoagulation needle (0.5mm) was inserted into the pterygoid foramen to block the bilateral vertebral arteries by electrocoagulation. The incision was sutured and the rats were back to the cage when they were awake. Twenty-four hours later, the same anesthesia was applied. An electrode was inserted under the skull and the reference electrode was placed under the skin of ear to monitor the changes of EEG. The disappearance of righting reflex, existence of spontaneous breathing, and the brain wave becoming flattening were the sign of global brain ischemia in this group. In the Sham operation group, the bilateral vertebral arteries were not occluded, and the bilateral common carotid arteries were not blocked. The rest of the operations were the same as the ischemia group.

### 2.5. Nissl Staining

Each group of paraffin sections was dewaxed to water according to the instructions of the Nissl's staining. The changes of rat cortical neurons after mounting were observed under a microscope.

### 2.6. Isolated Vessels Experiments [16]

Rats were anaesthetized as mentioned above. The brain was quickly taken out. The basilar artery was carefully dissected in the precooled physiological salt solution (PSS) and cut in rings that were placed into the vessel perfusion bath. Glass microtubules were sheathed at the two ends of the vascular ring and fixed with 10-0 surgical ties. In the 37°C PSS (95%O_2_+5%CO_2_, pH 7.4), the inner cerebral artery cavity was perfused by arterial pressure perfusion (150 *μ*l.min^−1^, 11.305 kPa).

Rat CBA segments were coincubated with PSS solution containing L-NAME (a NOS inhibitor, 3×10^−5^ mol/L) and indomethacin (a PGI_2_ inhibitor, 10^−5^ mol/L) or HC-067047 (a specific TRPV4 inhibitor, 10 *μ*mol/L), TRAM-34 (a specific IK_Ca_ inhibitor, 1 *μ*mol/L), and Apamin (a specific SK_Ca_ inhibitor, 0.5 *μ*mol/L) for 30 min. To determine the vasorelaxation effect, the CBA segments were precontracted by adding 30 mmol/L KCl to the luminal perfusate, and once the sustained constriction was obtained, TFR (11~2700 mg/L) was added cumulatively to induce a concentration-dependent vasodilation. The diameter of the basilar artery of the brain was observed and measured under stereo microscope, and the changes of the diameter of the basilar artery were measured. The percentage of dilatation = [(D_x_ - D_min_)/ (D_max_ -D_min_)] · 100%, here D_x_ indicates the diameter of the blood vessel after adding the corresponding test drug, D_min_ stands for the diameter of the blood vessel after adding KCl, and D_max_ represents the vessel diameter at 1 h after vascular equilibrium.

### 2.7. Membrane Potential Recording in the CBA Segments with Intact Endothelium [16]

The rats were quickly decapitated and the basilar artery was taken out. The vessel segments were cut longitudinally under an inverted microscope and fixed in the perfusion tank. Care was taken to preserve intact endothelium in these vessels. The inner surface of the blood vessel was infused with physiological salt solution (95% O_2_ + 5% CO_2_ mixed gas, 37°C) and incubated for 1 h. The corresponding solvent or drug was added to the perfusion fluid and incubated for 0.5 h. The glass microelectrode (resistance 30-50 MΩ, 3 mol·L^−1^ KCl) was pushed to the blood vessel surface with a micromanipulator under a stereo microscope and the cells were punctured. The signal was amplified by the microelectrode amplifier and transmitted to the Powerlab/4sp computer signal acquisition system to record the resting membrane potential of vascular smooth muscle cells. Hyperpolarization of the smooth muscle cell membrane was observed when the negative value of the resting membrane potential further increases.

### 2.8. Whole-Cell Patch Clamp Recording Experiment [16, 20]

Sprague-Dawley rats were sacrificed and their CBA was removed rapidly with global cerebral ischemia and reperfusion. The dissected CBA was immersed in PSS solution containing type II collagenase (1 mg/ml) and papain (0.5 mg/ml) and digested in a 37°C water bath for 35-40 min. After the digestion, the vessel was washed for four times using precooled PSS solution to eliminate the digestive enzymes. The digested tissue of CBA was separated into a single vascular smooth muscle cell and formed cell suspension by repeated blowing with different caliber suction tubes. Cell suspension was carefully sucked out and dripped onto glass coverslips with incubation for 30-45 minutes. After the cells adhered to the wall, the bath solution for recording K_Ca_ was added to the culture dish, which contained (in mM) NaCl 140, MgCl_2_ 1, H-HEPES 5, CaCl_2_ 1, KCl 5, and glucose 10 and was adjusted to a pH of 7.4 with NaOH. The whole-cell patch clamp recording experiments were carried out immediately. K^+^ current was recorded using whole-cell voltage-clamp recording mode (EPC-10 amplifier, membrane potential clamp at -60 mV). The patch pipettes with a tip diameter of 1-5 *μ*m were drawn out of glass microelectrode (resistance 3 to 5 MΩ) by using P-97-type microelectrode puller instrument from Sutter Instrument Company, USA. The intracellular patch pipette filling solution contained (in mM): K-gluconate 105, MgCl_2_ 1, KCl l30, H-HEPES 10, CaCl_2_ 2.1, and Na_2_ATP 5 (pH adjusted to 7.2 with NaOH). Using a step-square-wave pulse protocol, i.e., voltage from -60 mV to 100 mV, with a 10 mV step depolarization test for 500 ms, a typical outward current was recorded. The Igor 5 software was used for analyzing experimental results and the current density (pA/pF) was used for recording values of the current.

### 2.9. Western Blot Experiment

A total of 72 male SD rats were randomly divided into 9 groups: Sham (NS), Model (NS), TFR (100mg/kg), TFR+HC-067047 (100mg/kg+10mg/kg), HC-067047(10mg/kg), TFR+TRAM-34 (100mg/kg+0.5mg/kg), TRAM-34 (0.5mg/kg), TFR+Apamin (100mg/kg+0.3mg/kg), and Apamin (0.3mg/kg). In each group the above drug/chemicals were injected via tail vein 30 min before ischemia, and all rats were killed after ischemia for 25 min followed by 2 h of reperfusion. The endothelial cells from CBA in rats were isolated and purified by means of utilizing magnetic activated cell sorting (MACS) by the method performed as described in detail elsewhere by us [16] and by others [21]. The protein of the endothelial cells from CBA was extracted using cold lysis method, and the protein concentration was determined according to the BCA protein concentration kit. The anti-TRPV4, anti-IKca, and anti-SKca antibodies were applied to determine the protein expression in each group. Gapdh gene, highly expressed in almost all tissues and widely used as an internal reference for western blot protein standardization, was used as internal reference to compare the protein content.

### 2.10. Laser Scanning Confocal Experiment

Male SD rats were randomly divided into 6 groups (n= 8 for each), i.e., Sham (NS), Model (NS), TFR (100 mg/kg), TFR+HC-067047 (100 mg/kg+10 mg/kg), TFR+TRAM-34 (100 mg/kg+0.5 mg/kg), and TFR+ Apamin (100 mg/kg+0.3 mg/kg). The method of administration* in vivo* is the same as that of western blot experiment. In each group the above drugs/chemicals were injected via tail vein 30 min before ischemia, and all rats were killed after ischemia for 25 min followed by 2 h of reperfusion. The rats were anesthetized again and decapitated. The brain was removed and immersed in precooled physiological salt solution (PSS). The vessels were cut into small pieces and placed in 1 ml digestive enzyme solution (Collagenase: 2 mg/ml, Papain: 9 mg/ml, BSA: 5 mg/ml, DTT: 1.75 mg/ml) at 37°C for 45 min with shaking slightly every 15 minutes. At the end of the digestion the digestive enzymes were discarded and replaced with 0.5 ml precooled PSS. Each group of vascular smooth muscle cells was washed with D-hanks solution and then 2 ml cell culture medium was added. A proper amount of Fluo-3/ AM was added to make the final concentration of 2.5 g/ml. The vascular smooth muscle cells were incubated at 37°C for 40 min and then the Fluo-3/AM loading solution was removed. The fluorescent dye was washed by D-hanks solution. Fresh medium (200 *μ*l) was add and the sample was kept in dark for 15 min in order to promote the hydrolysis of intracellular esterification probe. The fluorescence intensity of Fluo-3 in the cell was observed by confocal laser scanning microscope, and the mean fluorescence intensity of individual cells in each group was analyzed by Image-Pro plus image analysis software.

### 2.11. Statistical Method

All data are expressed as the mean ± SEM. One-way analysis of variance (ANOVA) with Bonferroni's post hoc test was used for comparison among multiple groups. Unpaired t-test was used for comparison between two groups. To test the homogeneity of variance, SNK-q test method was used for homogeneity or Tamhane's T2 test method was used if not. SPSS 20.0 was used for statistical analysis.* P* <0.05 was accepted as statistically significant.

## 3. Results

### 3.1. Effects of HC-067047 and Other Blockers on the Improvement of Pathologic Injury of Brain Tissue by TFR in CIR Rats

**Figure 1:**
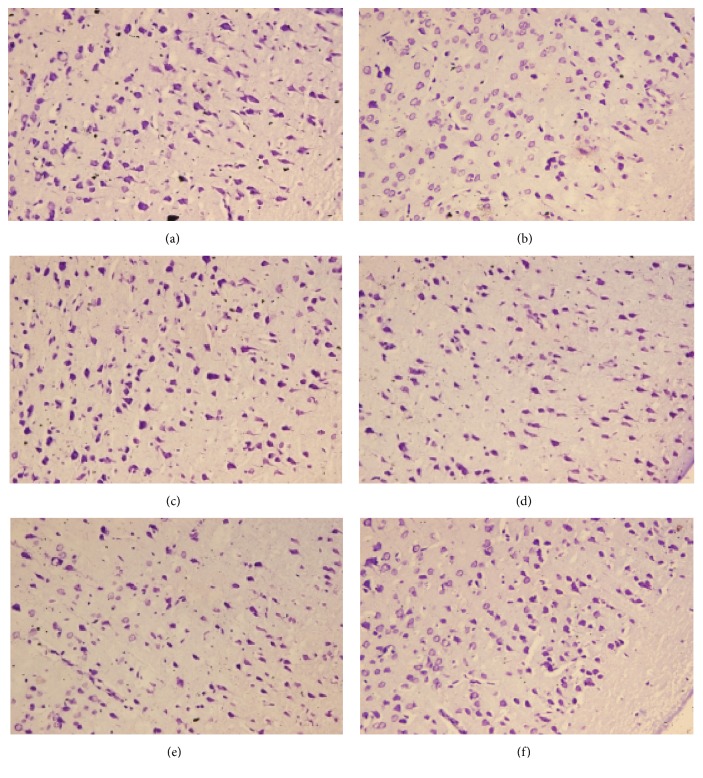
**Effects of HC-067047 and other blockers on the improvement of pathologic injury of brain tissue in CIR rats by TFR (Nissl staining, x 400)**. (a) Sham; (b) Ischemic; (c) TFR; (d) TFR+HC-067047; (e) TFR+Apamin; (f) TFR+TRAM-34.

Nissl staining results showed that, compared with Sham Group, the pyramidal cells in the cortex of ischemia group were sparse and disordered, and there were vacuoles of pyramidal cells or irregular-shaped cells with the number of pyramidal cells decreased. Further, there was empty staining or light staining. Compared with Ischemic Group, the vacuoles of pyramidal cells in the TFR group were reduced, the arrangement of pyramidal cells was neat, and the structure was more compact. In addition, the pathological changes of cortical neurons in the TFR+HC-067047 group, TFR+Apamin group or TFR +TRAM-34 group were also improved, although the phenomenon of decrease in cell number and the empty staining or light staining still existed in comparison to the TFR group. These results suggest that TFR has a protective effect on improving the pathological injury of cerebral cortex in rats with global cerebral ischemia and the effect is related to TRPV4, SK_ca_, and IK_ca_ channels. (Figure 1)

### 3.2. Effect of Inhibitors of TRPV4, SKca, and IKca on EDHF-Mediated Dilation and Hyperpolarization Induced by TFR in the CBA

**Figure 2:**
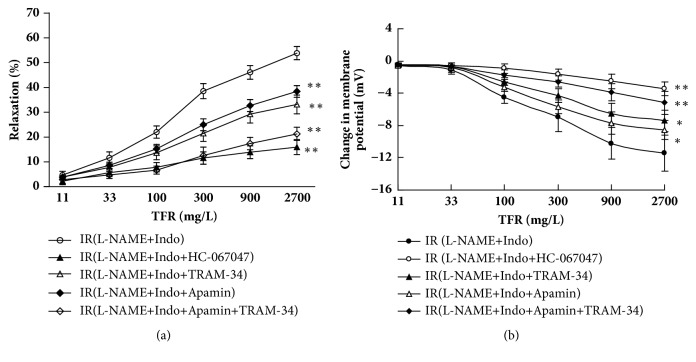
**Effect of inhibitors of TRPV4, SKca, and IKca on EDHF-mediated dilation and hyperpolarization induced by TFR (n=6 in each group).** L-NAME (an inhibitor of nitric oxide synthase), 30 *μ*mol/L; Indo (indomethacin, an inhibitor of cyclooxygenase), 10 *μ*mol/L. (a) Effect of inhibitors of TRPV4 (HC-067047), SKca (Apamin), IKca (TRAM-34) on EDHF-mediated dilation induced by TFR. (b) Effect of inhibitors of TRPV4, SKca, and IKca on EDHF-mediated hyperpolarization of cerebral vascular smooth muscle cells induced by TFR. *∗P*<0.05; ^*∗∗*^*P*<0.01 versus IR (L-NAME+Indo). Comparisons were made by unpaired Student's t-test.

As shown in Figure 2, CIR rats were pretreated with Indo (10 *μ*mol·L^−1^) and L-NAME (30 *μ*mol·L^−1^) for 30 min, TFR induced non-NO and non-PGI_2_ (EDHF) dilatation (the percentage of maximal relaxation, E_max_: 53.83±2.65%), and smooth muscle cell hyperpolarization (the change of membrane potential: -11.41±2.25 mV). Vehicle did not show any effect on either dilatation or hyperpolarization. In the CBA groups treated with inhibitors, the relaxation and hyperpolarization were all significantly reduced in comparison to the control (treated with Indo and L-NAME as mentioned above). The relaxation and hyperpolarization (change of membrane potential) were 15.98±3.01% versus control,* P* <0.01 and -3.47±0.83 mV versus control,* P* <0.01 in the group treated with TRPV4 inhibitor HC-067047 (10 *μ*mol·L^−1^), 38.39±2.38% versus control,* P* <0.01 and -8.55±1.14 mV versus control,* P* <0.05 in the group treated with SK_Ca_ inhibitor Apamin (0.05 *μ*mol·L^−1^), 33.17±3.80% versus control,* P* <0.01 and -7.43±1.32 mV versus control,* P* <0.05 in the group treated with IK_ca_ inhibitor TRAM-34 (1 *μ*mol·L^−1^), and 21.27±2.65% versus control,* P* <0.01 and -5.16±1.43 mV versus control,* P* <0.01) in the group treated with Apamin plus TRAM-34 (ANOVA and Bonferroni's post hoc test for the above comparisons).

These vessels were endothelium-intact and therefore the results suggest that the EDHF-mediated dilation and hyperpolarization induced by TFR in the CBA of CIR rats is related to TRPV4, SK_Ca_, and IK_Ca_ channels.

### 3.3. Effects of TRAM-34 and Apamin on Calcium Dependent Potassium Currents Induced by TFR in the Smooth Muscle Cells of the CBA

**Figure 3:**
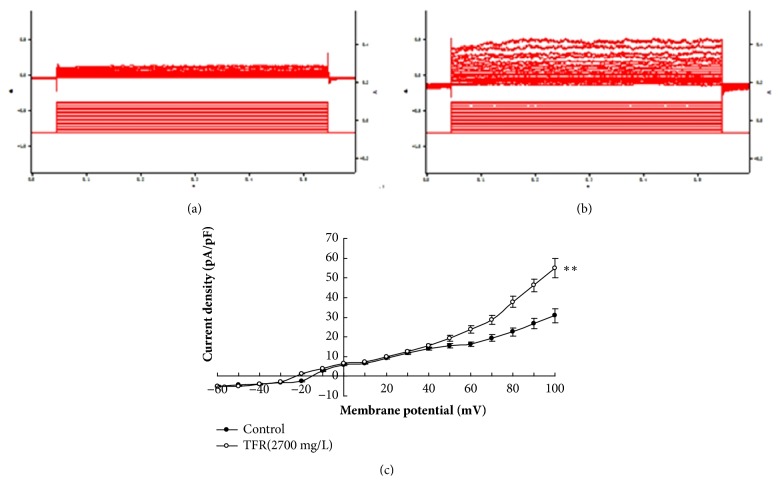
**TFR induced outward currents in CBA smooth muscle cells of CIR rats (n=6 in each group).** (a) Control group; (b) TFR group; (c) Current-voltage curve. ^∗∗^*P*<0.01* versus* Control (unpaired t-test).

**Figure 4:**
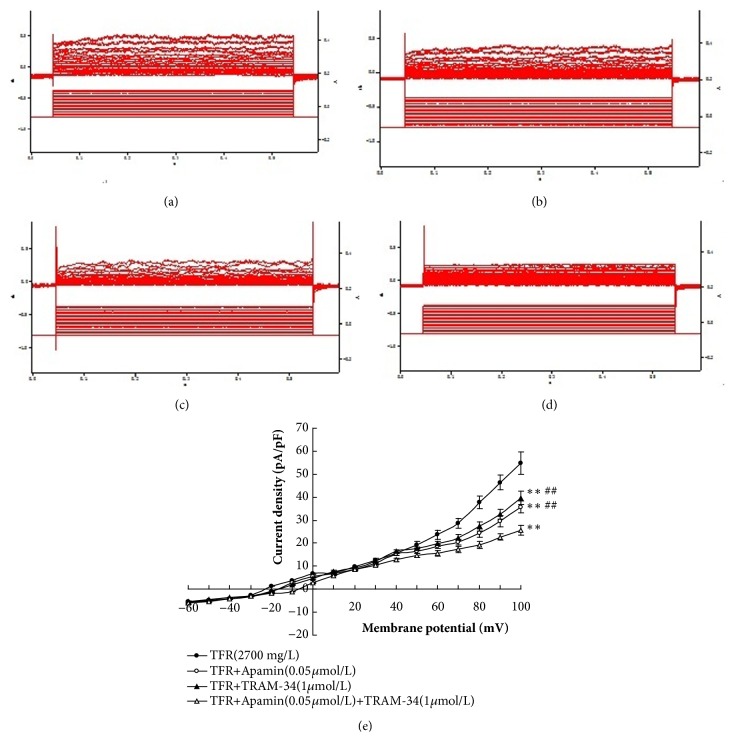
**Effects of inhibitors of the SKca and IKca channels on TFR induced outward current (n=6 in each group).** ^∗∗^ *P*<0.01* versus *TFR (2700 mg/L); ^##^*P*<0.01* versus *CIR+TFR+Apamin (0.05*μ*mol/L)+TRAM-34 (1*μ*mol/L). (a) TFR induced outward currents in the smooth muscle cell of CBA in CIR rats. (b) Effects of SK_Ca_ channel blocker Apamin on outward currents induced by TFR. (c) Effects of IK_Ca_ channel blockers TRAM-34 on outward currents induced by TFR. (d) Effects of Apamin plus TRAM-34 on outward currents induced by TFR. (e) Current-voltage curve.

TFR (2700 mg·L^−1^) was added to the extracellular fluid of CBA smooth muscle cells from CIR rats; an outward current was clearly elicited (pA/pF: 54.9±4.9,* P* <0.01, Figure 3). Adding of TRAM-34 (1 *μ*mol·L^−1^) (39.7±2.9 versus 54.9±4.9,* P* <0.01, unpaired t-test) or Apamin (0.05 *μ*mol·L^−1^) (35.7±2.6 versus 54.9±4.9,* P* <0.01) into the fluid significantly attenuated the increased outward current density induced by TFR (2700 mg·L^−1^), and the combination of TRAM-34 and Apamin had an additive effect (25.6±2.2 versus 54.9±4.9,* P* <0.01, ANOVA and Bonferroni's post hoc test; Figure 4). These results suggest that the TFR induced outward currents in the smooth muscle cell of CBA in CIR rats are related to the opening of SK_ca_ and IK_ca_ channels.

### 3.4. Effects of TFR and Channel Inhibitors on the Protein Expression of the TRPV4, IK_ca_, and SK_ca_ Channels of the Endothelial Cells from CBA in CIR Rats

**Figure 5:**
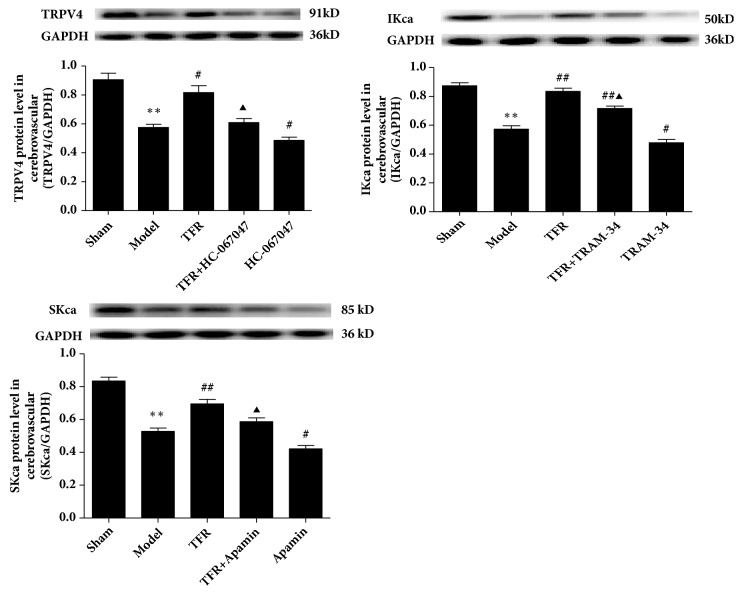
**Effect of TFR and each channel blocker on TRPV4, IKca, SKca protein expression levels of** the endothelial cells from** cerebral basilar arteries in rats of ischemia/reperfusion injury** (model)** (n=4 in each group).**^∗∗^*P*<0.01 versus Sham; ^#^*P*<0.05, ^##^*P*<0.01 versus Model (Ischemic); ^▲^*P*<0.05 versus TFR.

Figure 5 shows that the expression of the protein of TRPV4, IK_ca_, and SK_ca_ of the endothelial cells from CBA was significantly decreased in CIR rats compared to the Sham rats (TRPV4: 0.58±0.04 versus 0.91±0.08; IK_ca_: 0.57±0.04 versus 0.87±0.04; SK_ca_: 0.53±0.03 versus 0.83±0.04,* P*<0.01), whereas TFR-treatment significantly increased the protein expression of these channels. The effect of TFR was attenuated by either HC-067047 (0.61±0.05 versus 0.82±0.08,* P*<0.05), TRAM-34 (0.72±0.03 versus 0.84±0.04,* P*<0.05), or Apamin (0.59±0.04 versus 0.70±0.05,* P*<0.05, ANOVA and Bonferroni's post hoc test for the above comparisons).

### 3.5. Effect of HC-067047 on the Protein Expression of IKca and SKca Channels of the Endothelial Cells from CBA in CIR Rats

**Figure 6:**
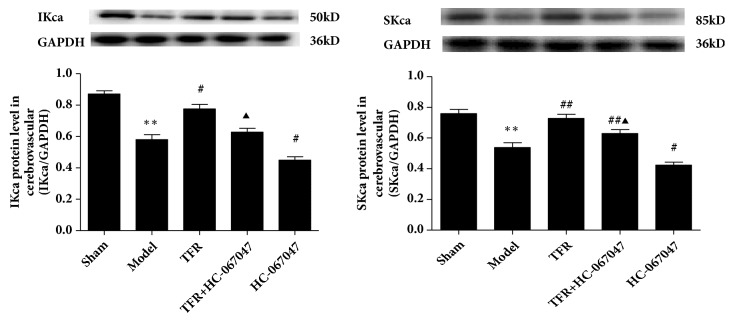
Effect of HC-067047 on the protein expression of IKca and SKca channels of protein expression levels of the endothelial cells from cerebral basilar arteries in rats of ischemia/reperfusion injury (model) (n=4 in each group). ^∗∗^*P*<0.01 versus Sham; ^#^*P*<0.05, ^##^*P*<0.01 versus Model (Ischemic); ^▲^*P*<0.05 versus TFR.

Figure 6 shows that the protein expression of IKca and SKca of the endothelial cells from CBA was significantly reduced by CIR and increased by TFR. The increase of the protein by TFR was significantly attenuated by HC-067047 (IKca: 0.78±0.05 versus 0.63±0.04; SKca: 0.73±0.05 versus 0.65±0.04,* p*<0.05; ANOVA and Bonferroni's post hoc test for the above comparison), showing that inhibition of TRPV4 channel downregulates the increased expression of SKca and IKca proteins induced by TFR in the CBA in CIR rats.

### 3.6. Effect of TFR and Channel Blockers on Ca^2+^ Concentration of CBA in CIR Rats

**Figure 7:**
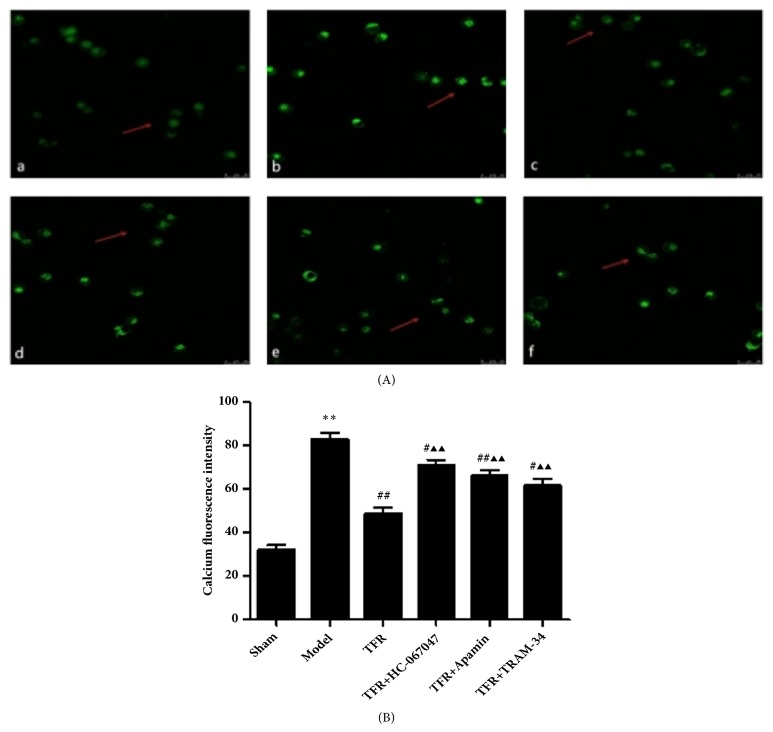
**Laser scanning confocal analysis of Ca** ^**2+**^ **fluorescence intensity in rat cerebral basilar artery smooth muscle cells**. In each group the drugs/chemicals were injected via tail vein 30 min before ischemia, and all rats were killed after ischemia for 25 min followed by 2 h of reperfusion. (A) Cells pretreated with Fluo-3/AM. (a) Ca^2+^ fluorescence intensity in Sham group; (b) Ca^2+^ fluorescence intensity in Ischemic group; (c) Ca^2+^ fluorescence intensity in TFR group; (d) Ca^2+^ fluorescence intensity in TFR+HC-067047 group; (e) Ca^2+^ fluorescence intensity in TFR+Apamin group; (f) Ca^2+^ fluorescence intensity in TFR+TRAM-34 group. (B) Effect of TFR and each channel blocker on Ca^2+^ fluorescence intensity of cerebral basilar artery smooth muscle cells in rats of ischemia/reperfusion injury. ^∗^*P* <0.01 versus Sham; ^#^*P*<0.05, ^##^*P*<0.01 versus Model (Ischemic); ^▲▲^*P*<0.01 versus TFR.

The mean fluorescence intensity of Ca^2 +^ in the smooth muscle cells of CBA in the Sham Group was 32.02 ± 5.93. It was significantly increased in Ischemic group that was markedly decreased by TFR (82.78 ±7.36 versus 48.65±7.46 in control,* P*<0.01). The effect of TFR was attenuated by either HC-067047 (70.70±6.66 versus control,* P*<0.01), TRAM-34(61.60±7.27 versus control,* P*<0.01), or Apamin (66.65±4.74 versus control,* P*<0.01, ANOVA and Bonferroni's post hoc test for the above comparison; Figures 7(A) and 7(B)).

## 4. Discussion

The present study for the first time demonstrated that in the CBA in the CIR rats. (1) The protective effect of TFR on ischemic cerebrovascular injury may be related to the activation of the TRPV4 in the vascular wall by increasing its expression and activity as well as reducing Ca^2+^ concentration. (2) The TFR induced EDHF-mediated relaxation and hyperpolarization is related to the SKca and IKca channels. (3) Activation of TRPV4 may be linked to the opening of endothelial IKca/SKca channels to mediate the EDHF-like responses.

It is well known that endothelium-dependent dilatation is mainly mediated by NO, PGI_2_, and EDHF [20]. EDHF is an important modulator in regulating cerebral blood flow during normal physiological states and plays an even greater role under pathological conditions such as hypoxia, acidosis, and organ ischemia [21].

TFR is the active extract from the flowers of Rhododendron and has been found to have anti-inflammatory, analgesic, and antispasmodic role [22]. Our previous studies have shown that TFR plays a protective role against cerebral ischemia-reperfusion injury by activating EDHF-mediated cerebrovascular relaxation [16, 17].

TRP channels are interacted with the release of NO as we previously demonstrated [23]. Studies have shown that Ca^2+^-entry mediated by the endothelial TRPV4 is involved in the synthesis of nitric oxide [24] and in EDHF signaling [25, 26], and that activation of endothelial TRPV4 promotes the opening of SK_Ca_ and IK_Ca_ channels [27], expressed in ECs [28]. Our findings are in accordance with this.

In addition, we have demonstrated the modulating role of IKca and SKca channels in homocysteine-induced endothelial dysfunction [29]. It was also demonstrated that inhibition of SK_ca_ expression depolarizes both endothelial cells and smooth muscle cells, reduces the diameter of resistance vessels, and raises blood pressure, while restoration its expression may reverse this phenomenon [30]. Further, the destruction of IK_Ca_ expression significantly decreases EDHF-mediated reaction and reduces ACh-mediated hyperpolarization of endothelial cells and smooth muscle cells that is linked with reduced vasodilation. In the experiment of IK_Ca_ and SK_Ca_ double knockout mouse, simultaneous deletion of both genes could lead to more severe damage [31, 32].

In the present study, we further explored the relationship among TRPV4, SKca and IKca channels and EDHF-mediated effects induced by TFR on anti-ischemic brain injury in CIR rats. Our results of Nissl staining showed that the pathological injury of cerebral cortex in CIR rats was significantly improved with treatment of TFR and this effect was inhibited by either highly selective blocker of TRPV4 channel HC-067047[33], SK_Ca_ channel-specific blocker Apamin, or IK_Ca_ channel-specific blocker TRAM-34 [34]. These results suggest that TFR has a favorable effect on cerebral cortical injury in CIR rats and the effect is associated with TRPV4, SKca, and IKca channels.

In our* in vitro* vasodilation and cell membrane potential recording experiments, we found that, after excluding the vasodilation of PGI_2_ and NO by applying cyclooxygenase inhibitor Indo and NO synthase inhibitor L-NAME, TFR induced and EDHF-mediated relaxation and hyperpolarization of CBA in CIR rats were blocked by HC-067047 or Apamin or TRAM-34. This is consistent with a previous study reporting that the effect of NO and EDHF was weakened in ACh-induced vasodilation in TRPV4 knockout mice [26]. These vessels were endothelium-intact and therefore the results suggest that the EDHF-mediated dilation and hyperpolarization induced by TFR in the CBA of CIR rats are related to TRPV4, SK_Ca_, and IK_Ca_ channels. Because TRPV4 is located in both endothelium and smooth muscle, we could not distinguish whether the opening of TRPV4 is due to opening of endothelial TRPV4 or opening of smooth muscle TRPV4, perhaps both. However, the opening of IK_ca_ and SK_ca_ by TFR demonstrated in Figure 2(b) is most likely due to the opening of IK_ca_ and SK_ca_ in the endothelial cell (because IK_ca_ and SK_ca_ are located mainly in the endothelial cell) that is one of the major mechanisms for the EDHF-mediated hyperpolarization in the smooth muscle cell as well-known [7, 8, 13].

Next, we observed whether TFR could induce calcium dependent potassium currents in CBA smooth muscle cells of CIR rats and the effects of blocking agents TRAM-34 or Apamin. We found that TFR elicited an outward current in acutely isolated CBA smooth muscle cells from CIR rat and that the current was visibly eliminated by either TRAM-34 or Apamin. The combination of these two inhibitors (TRAM-34 and Apamin) had even more significant effect. These results indicate that the effects of TFR involve the opening of the SK_Ca_ and IK_Ca_ channels.

Importantly, we also observed the effect of TFR and channel blockers on the expression of the endothelial TRPV4, SKca, and IKca proteins in cerebral vessels of the CIR rats. The results showed that the expression of the endothelial TRPV4, SK_Ca_, and IK_Ca_ channels in rat CBA was significantly increased by administration of TFR but decreased by HC-067047, Apamin, and TRAM-34 (Figures 5 and 6). These results provide direct evidence that TFR upregulates the expression of the endothelial TRPV4, SK_Ca_, and IK_Ca_ proteins in the CBA of CIR rats.

In order to further investigate the relationship between TRPV4 and SKca/IKca channels in the role of TFR in anti-ischemic brain injury, we detected the expression of the endothelial SKca and IKca proteins in cerebral vascular endothelial cells of CIR rats by blocking TRPV4 channel. The results showed that the expression of SK_Ca_ and IK_Ca_ proteins upregulated by TFR was significantly reduced by HC-067047 (Figure 6), suggesting that TFR upregulates the expression of the endothelial SK_Ca_/IK_Ca_ proteins in CBA by activating TRPV4.

Further, we found that the mean fluorescence intensity of Ca^2+^ in rat cerebral smooth muscle cells was markedly reduced after administration of TFR and the effect was abolished by HC-067047, Apamin, or TRAM-34 in the* in vivo *experiments, suggesting the role of the endothelium in the relaxation/hyperpolarization. This result is in accordance with the relaxation/hyperpolarization as well as protein expression experiments in this study. It should be thought that opening of TRPV4 channels in smooth muscle cells should allow Ca^2+^ influx and increase the intracellular Ca^2+^ ([Ca^2+^]i) intensity if this is the ONLY mechanism. The explanation to the reduction of [Ca^2+^]i by TFR is probably due to the complex effect of TFR in vessels. As discussed above, TFR activates the TRPV4 channel in the smooth muscle cell that increases calcium influx. Simultaneously, TFR opens TRPV4 in the endothelial cell that activates IK_Ca_ and SK_Ca_ channels of the endothelial cell (Figures 5 and 6). In addition, it is possible that TFR may also directly open the IK_Ca_ and SK_Ca_ channels of the endothelial cell. These effects hyperpolarize the endothelial membrane and subsequently hyperpolarize the smooth muscle cell membrane (Figures 2 and 3; [8, 13]) and open BK_Ca_ channel of the smooth muscle cell [8, 13], which blocks the voltage-dependent calcium channels of the smooth muscle cell[8, 13] and reduces the [Ca^2+^]i. Further, there is a TRPV4-dependent pathway in the activation of BK_Ca_ channels in the vascular smooth muscle cell [35] and the activation of TRPV4 in the smooth muscle cell in CBA may be linked with the activation of BK_Ca_ channel. The latter blocks the voltage-dependent calcium channel and relaxes the vessel [7, 8, 13]. The net effect of the above mechanisms is reduction of [Ca^2+^]i that finally relaxes/dilates the smooth muscle cell.

Taken together, our study demonstrates that TFR upregulates the expression of the endothelial SK_Ca_/IK_Ca_ proteins in CBA by activating TRPV4. As shown in the Figures 5 and 6, in the endothelium, the activation of TRPV4 channels opens the SK_Ca_/IK_Ca_ channels that leads to EDHF-mediated hyperpolarization and relaxation of the smooth muscle cell. Further, the activation of TRPV4 in the smooth muscle cell in CBA may be linked with the activation of BK_Ca_ channel through a TRPV4-dependent pathway [35]. The activation of BK_Ca_ channel blocks the voltage-dependent calcium channel and relaxes the vessel [7, 8, 13]. Therefore, the mechanism of the protective effect of TFR in CBA of CIR rats is related to the TRPV4 channel-associated hyperpolarization and relaxation.

## 5. Conclusion

We conclude that in the CBA of the CIR rats the protective effect of TFR on ischemic cerebrovascular injury may be related to the activation of the TRPV4 in both endothelium and smooth muscle by increasing its expression and activity. As shown in protein expression results in the endothelial cells (Figures 5 and 6), the activation of TRPV4 channel in the endothelium may be linked to the opening of endothelial IKca/SKca channels that induces EDHF-mediated relaxation and hyperpolarization in the smooth muscle cell. In addition, the activation of TRPV4 in the smooth muscle cell in CBA may be linked with the activation of BK_Ca_ channel through a TRPV4-dependent pathway, reduce Ca^2+^ concentration in the cell, and relaxe the vessel. These findings may form a new therapeutic target for protection of ischemic brain injury and facilitate the use of Chinese medicine in brain protection.

## Data Availability

The data used to support the findings of this study are available from the corresponding author upon request.

## Abbreviation

ACh: Acetylcholine
BCA: Bicinchoninic acid
CIR: Cerebral ischemia-reperfusion
CBA: Cerebral basal artery
EDHF: Endothelium-dependent hyperpolarizing factor
EEG: Electroencephalograph
GAPDH: Glyceraldehyde 3-phosphate dehydrogenase
IR: Ischemia/reperfusion injury
PGI_2_: Prostacyclin
NO: Nitric oxide
PSS: Precooled physiological salt solution
RR: Ruthenium red
SD rats: Sprague-Dawley rats
TRP: Transient receptor potential
TRPV: TRP vanilloid channel
TFR: Total flavonoids of Rhododendron.
